# Incisor torque expression characteristics in two passive self-ligating brackets placed at different heights. A finite element investigation

**DOI:** 10.1016/j.jobcr.2024.01.003

**Published:** 2024-01-15

**Authors:** R.P Bernisha, Gyanda Mishra, G. Pradeep Raj, Prasad Chitra

**Affiliations:** Department of Orthodontics and Dentofacial Orthopaedics, Army College of Dental Sciences, Secunderabad, Telangana, 500087, India

**Keywords:** Torque, Passive self-ligation, Orthodontic brackets, Incisor, Bracket height

## Abstract

**Objective:**

This study investigated torque expression in maxillary incisors using two passive self-ligating bracket types (Damon Q and Pitts 21) placed at different heights using the Finite element method.

**Materials and methods:**

Two passive self-ligating brackets, Damon Q (Ormco, USA) and Pitts 21 (OC Orthodontics, USA) were 3D modeled using micro-computed tomography. Damon Q (0.022ˮ x 0.028″ slot size) and Pitts 21 (0.021ˮ x 0.021″ slot size) brackets were placed on a maxillary central incisor at predetermined vertical heights. Arch wires of size 0.019ˮ x 0.025″ stainless steel (Damon Q) and 0.020ˮ x 0.020” Titanium Molybdenum (Pitts 21) were placed in the bracket slots.

**Results:**

Pitts 21 brackets showed higher torquing moments at all bonding heights as compared to Damon Q brackets. The minimum torquing moment was 9.03Nmm at 5 mm for Damon Q and the maximum torquing moment was 14.92Nmm for Pitts 21 at a bracket bonding height of 8 mm. Total deformation for Pitts 21 at a height of 5 mm from the incisal edge was 0.61 × 10^−6^mm as compared to that of Damon Q which was 0.41 × 10^−6^mm. Lowest Von Mises stress values were at 27.07 MPa in Damon Q brackets at a bracket height of 5 mm from the incisal edge. Highest Von Mises stress values were 36.80 MPa for Pitts 21 brackets at a bracket height of 8 mm from the incisal edge.

**Conclusion:**

Pitts 21 brackets exhibited superior torquing characteristics compared to Damon Q. Total deformation in Pitts 21 was higher than Damon Q at all tested bracket bonding heights.

## Graphical abstract

**Figure:**
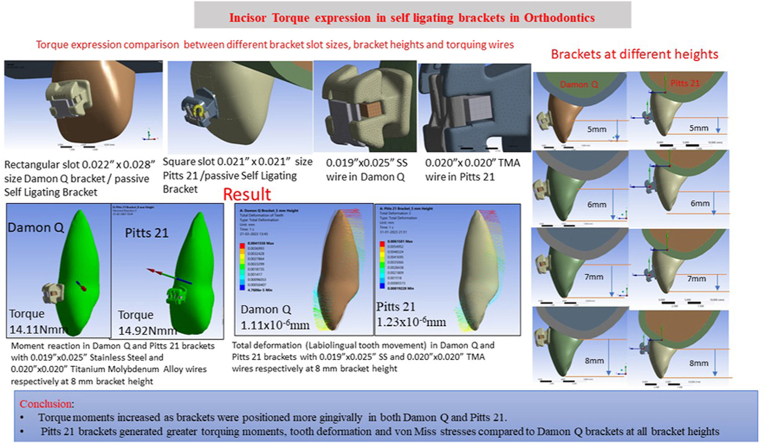

## 1 Introduction

Torque can be defined as the twisting of a structure about its longitudinal axis, resulting in an angle of twist. In orthodontics, it represents the labiolingual or buccolingual crown/root inclination of a tooth. The extent of torque expression depends on a multitude of factors that include stiffness or wire resilience, wire size and cross-section, edge bevel and manufacturer tolerances, engagement angle of the wire in a bracket slot, as well as bracket placement position on a tooth.^1^

Angle introduced the Edgewise system based on three-dimensional tooth control, which was obtained by engaging a rectangular wire into a rectangular slot bracket. Torque was achieved using 3rd order bends placed in archwires.^2^ As a rectangular wire is twisted and inserted into a bracket slot, the opposite side of the wire contacts the slot, creating a couple and generating moments. Taking advantage of the control offered by the conventional Edgewise system, Andrews introduced the Straight Wire Appliance using tooth specific brackets with inbuilt in-out, tip and torque values.^3^ Steiner, in 1952, introduced a rectangular bracket with slot dimensions of 0.018”. The 0.022″ system showed mechanical advantages especially during sliding mechanics, where a 19″ x25″ wire was used. The 0.018” system showed superiority in the generation of a couple force when a 17˝ x 25˝ wire was engaged. The properties of the arch wire material (elastic modulus and elastic/super elastic behavior) as well as geometry (cross-section and slot dimensions) influence torque.^4^ Torque expression can be achieved by filling the bracket slot with increasing sizes of arch wires during treatment. However, in most situations, full dimension wires in rectangular slot brackets are rarely used, which means a significant percentage of torque built into the bracket is lost due to increased play between a smaller dimension arch wire and the bracket slot.^5^ It is essential to consider play since it directly affects tooth movement and force transmission to a tooth. Sliding tooth movements require some degree of play in order to minimize friction, whereas minimal play is required in situations where the wire requires to be deflected (torquing movements). Shima^5^ et al. assessed play between square and rectangular slot brackets by using arch wires of varying sizes. The results showed that horizontal play in square slots was significantly less than in rectangular slot brackets, which means that brackets with square slots can more effectively bring about tooth movement in the labiolingual direction as well as show better rotational control with the use of either round or square wires. Additionally, the horizontal and vertical play ratios for round and square wires in square slots were approximately 1.0 which indicates that three-dimensional tooth movement can be achieved with uniform play both in horizontal and vertical directions on the use of square slots.

Self-ligating brackets introduced by Stolzenberg in 1935 have gained popularity over the last few years due to claims of reduced friction and overall treatment time. Active and passive self-ligation brackets (SLBs) are available where load and moment expression can differ according to the clinical situation.^7^ Torque expression characteristics between conventional rectangular slot brackets and passive SLBs were assessed in a systematic review which showed superior torque control in the conventional bracket type.^8^ In contrast, active SLBs constantly apply forces on archwires in order to gain better tooth control but this leads to increased amounts of friction which could be a disadvantage.^9^ Optimal maxillary teeth labiolingual inclination is required for good treatment outcomes, smile esthetics and anterior guidance. Under torqued incisors can result in arch length and space discrepancies.^10^ Another study comparing torque expression characteristics between conventional and two different self-ligating bracket types provided evidence of lower torque expression in the self-ligating bracket types.^11^

Torque expression can also be influenced by bracket positioning on a tooth. A study by Meyer^12^ demonstrated a difference of 15° in torque expression with a 3 mm change in bracket position from the incisal edge. Miethke^13^ et al. observed a torque variation of between 10 and 15° with vertical discrepancy of 1 mm during bracket placement. Torque expression was increased with placement of brackets more gingival from the incisal edges. Clinically, the most commonly used bracket height is 5 mm. This feature can be exploited by clinicians to maintain or improve the smile arc for a patient. A consonant smile arc requires upright positioning of the maxillary incisors for better esthetics and function.

Use of self-ligating brackets has increased over the past few years with a significant number of clinicians claiming to use them in routine practice.^14^ The Pitts 21 (OC Orthodontics, Oregon, USA) square slot passive self-ligating system is a recent introduction utilizing square anterior bracket slots with square wires for increased engagement early in treatment.^15^ The benefits of the system according to the manufacturers are full engagement of archwires in anterior bracket slots early in treatment due to the square slot cross section (0.021˝ x 0.021˝) and use of specially designed heat activated 18˝ x 18˝, 20˝ x 20˝ Nickel Titanium and 20˝ x 20˝ Titanium Molybdenum Arch wires which permit early tip and torque control with light forces. Passive SLBs are used due to easier ligation, reduced friction, simple mechanics and supposedly reduced treatment times. However, their torque expression characteristics are uncertain due to the complex interactions between the wire and bracket slot which are difficult to determine clinically.^16^^,^^17^

Stress-strain measurements for varied materials including living tissues can be carried out using FEM (Finite element method). Complex bioengineering problems can be solved using this method.^18^ FEM can be utilized to simulate various treatment methods and observe responses without the need for animals or patients.^19^ However, FEM results may show a variation of up to 20% from real world conditions.^20^

The study aimed to determine efficacy of the Pitts 21 square slot passive SLB system in comparison to a rectangular slot passive SLB system (Damon Q, Ormco Corp, Glendora, USA) in order to assess torque expression characteristics in the maxillary central incisors for brackets placed at varying heights from the incisal edge. The brackets were scanned using micro-computed tomography (micro-CT) and later modeled using FEM. Square slot [0.018″ x 0.018’’] brackets have been developed for lingual^5^ and labial^6^ treatment in recent times to compensate for the deleterious effect of horizontal play. However, not much is known about the factors affecting torque expression in square slot brackets. The effect of arch wire material and dimension on torque expression in square brackets needs to be studied as there are no studies in literature evaluating the same. Also, with focus shifting towards aesthetic bracket placement, the Smile Arc Protection protocol advocates more gingival bracket bonding heights than conventional bonding. As the square Self Ligating brackets bonded as per SAP protocol are closer to the Centre of Resistance of the teeth, more tooth deformation [labio-lingual tooth movement] is postulated and the same needed to be investigated systematically. Finite element method allowed us to study the effects of multiple variables in a controlled scenario. Square slotted brackets allow for full slot engagement earlier during the treatment, thereby mitigating the effects of play and allowing early and effective torque control.^5^ The bracket slot size, bracket position and wire selection are the some of the most critical clinical variables that an orthodontist can control. Therefore, we aimed to compare the effect of bracket slot size, arch wire material and dimension and bracket height on torque expression in maxillary central incisor reflecting a more real –world simulation of clinical scenarios. The study had the following objectives:

A. To compare torque expression between a rectangular slot passive SLB (Damon Q) and square slot passive SLB (Pitts 21).
B. To assess the influence of bracket position on torque expression in both bracket types.
C. To compare the moments generated with engagement of a 0.019˝ x 0.025˝ stainless steel finishing wire into slots of Damon Q and 0.020˝ x 0.020˝ titanium molybdenum (TMA) wire into slots of Pitts 21 brackets.

## 2 Materials and methods

Two passive self-ligating brackets (Damon Q with a rectangular slot dimension of 0.022˝ x 0.028˝ and Pitts 21 with a square anterior slot dimension of 0.021˝ x 0.021˝) were mounted on strips of modelling wax with radiolucent carbon tape as part of the sample preparation process prior to scanning. The radiolucent tape prevented the formation of artifacts during the scanning process. The brackets were individually scanned using a micro computed tomography scanner (Skyscan 1271 Bruker, Belgium). The mounted samples were scanned in the Digital Imaging and Communications in Medicine format (DICOM) onto a compact drive for 45 min with a total of 1000 slices per scan. This permitted creation of high-quality models of the brackets with all surface details. Three -dimensional models of the upper left and right maxillary central incisors were also prepared. Finite element models of the scanned brackets were obtained and material properties including Young’s modulus and Poisson’s ratio were assigned for teeth, brackets and archwires used in the investigation using values obtained from a previous study^21^ as seen in Table 1.

**Table 1:** Average material property values.

S.No.	Linear -elastic material parameters	Young’s modulus of elasticity, E (MPa)^21^^,^^34^	Poisson’s Ratio
1.	Teeth	20,000	0.30
2.	Brackets	180,000	0.3
3.	Titanium Molybdenum Arch wire	86,000	0.3
4.	Stainless steel	1.93E +0.5	0.3
5.	Cortical bone	14.5	0.3
6.	Spongy bone	1.37	0.3

A 19˝ x 25˝ stainless steel finishing wire and a 20˝ x 20˝ TMA finishing wire were placed in the bracket slots of Damon Q and Pitts 21 respectively. 3D finite element models of both bracket types were based on scanned cross-sections obtained with the micro-CT scanner in DICOM format. The DICOM files were then imported into Mimics Research 21.0 software where the data underwent processing to convert the scans into stereolithography (STL) format. Masking was carried out by capturing the required region using a masking tool in Mimics software with use of Mimics Multiple Slice Edit segmentation tools to add or remove entities. The 3D models in STL format were then converted to solid models by exporting data to Ansys Space Claim R 22.0 software, where cleanup and model checking was performed. The solid models were imported into Solid Works 2021 where models were recreated according to the actual bracket dimensions.

A 3D computer aided drafting (CAD) model obtained from Turbo Squid (Turbo Squid, New Orleans, USA) served as the base for teeth. From this CAD model, modifications were made using Solid Works to alter tooth geometry according to dental anatomy literature. A 3D model of the maxillary right and left central incisor was constructed from the CAD model. Brackets were placed onto the teeth models at predetermined heights and a 0.019˝ x 0.25˝ SS and a 0.020˝ x 0.020˝ TMA archwire inserted into the bracket slots of a Damon Q and Pitts 21 bracket, respectively. The geometric models were then converted into finite element models. The elements could be one, two or three -dimensional and in various shapes and were connected at key points by nodes. A total of 1885715 elements were connected by 647,722 nodes for Damon Q and 333,210 elements were connected by 65,412 nodes for Pitts 21 brackets.

The mechanical properties assigned to the elements were isotropic (having physical properties of the same value when measured in different directions) and linear elastic (linear relationships between the components of stress and strain). Each structure was assigned a specific material property. Ansys Mechanical R22.0 was then used for importing models with 0% data loss. The software performed automatic meshing with defined material properties. In structural linear static Finite Element Analysis (FEA), the governing equation is typically derived from the equilibrium of forces. The fundamental equation is based on Newton’s second law and can be expressed as [K]. [U] = [F], where [K] is the stiffness matrix representing the stiffness of the structure, [U] is the vector of nodal displacements and [F] is the vector of applied nodal forces.

**Fig. 1:**
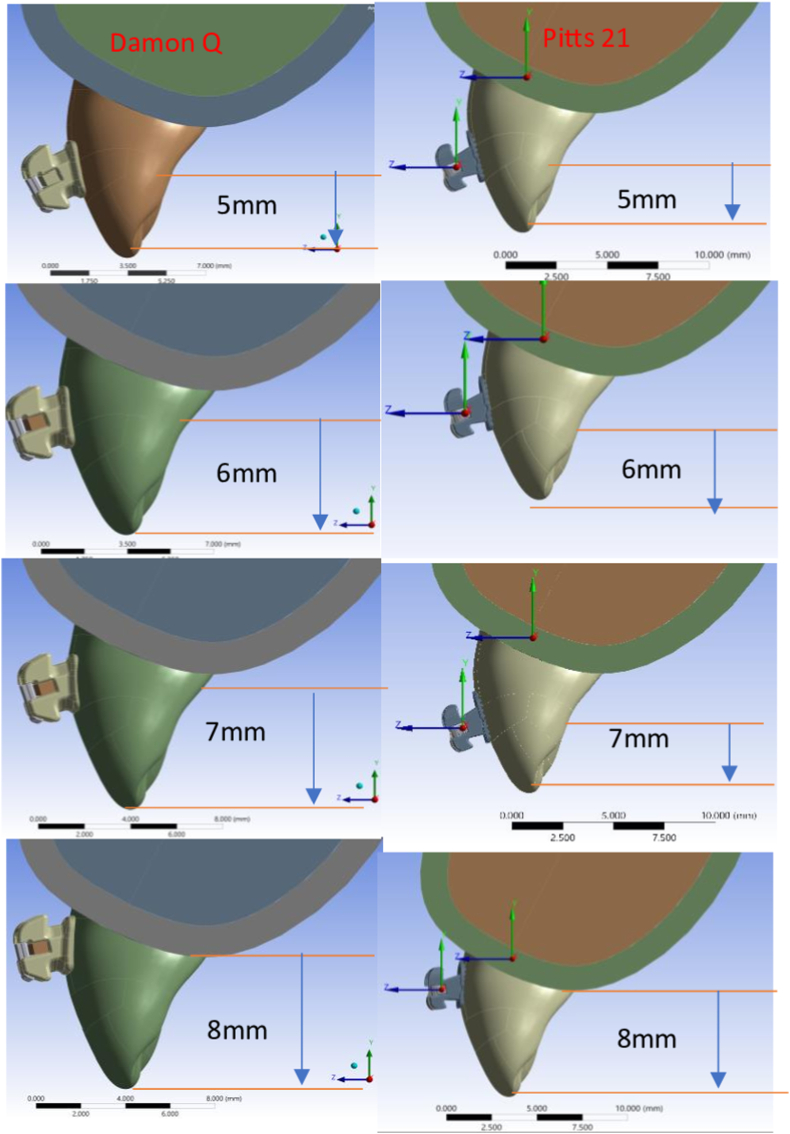
Damon Q and Pitts 21 brackets placed at different heights.

This equation is derived from the equilibrium of forces and the assumption that the deformation is small and follows Hooke’s Law. Solving this system of equations provides the nodal displacements and subsequently the stress and strain distributions within the structure.^22^ The brackets were placed at heights of 5 mm, 6 mm, 7 mm and 8 mm from the incisal edge in both bracket types and torque moments generated with use of full-size wires were recorded (Fig. 1). The results were evaluated from graphical and numerical formats obtained.

## 3 Results

The results are discussed under the following sections:

1. Comparison of incisor torque expression in Damon Q and Pitts 21 brackets placed at heights of 5, 6, 7 and 8 mm from the maxillary central incisor edge.
2. Total deformation (tooth movement) of the central incisors on engagement of a 0.019˝ x 0.025˝ SS wire into the bracket slot of a Damon Q bracket and engagement of a 0.020˝ x 0.020˝ TMA wire into the bracket slot of a Pitts 21 bracket.
3. Stress generated on central incisors, including roots with engagement of prescribed full-size wires into both tested bracket slots.

### 3.1 Comparison of incisor torque expression in Damon Q and Pitts 21 brackets placed at heights of 5, 6, 7 and 8 mm from the maxillary central incisor edge

The torquing moments generated in Damon Q and Pitts 21 brackets bonded on the maxillary central incisor at 5, 6, 7 and 8 mm heights from the incisal edges were calculated. The archwires used in the investigation were 19˝ x 25˝ SS for Damon Q and 20˝ x 20˝ TMA for Pitts 21. Torquing moments showed an increase for both bracket types tested as the bonding height was increased from 5 mm to 8 mm. Pitts 21 brackets showed higher torquing moments at all bonding heights as compared to Damon Q brackets. The minimum torquing moment was 9.03Nmm at 5 mm for Damon Q and the maximum torquing moment was 14.92Nmm for Pitts 21 at a bracket bonding height of 8 mm. Least torquing moments for both bracket types were observed at a bonding height of 5 mm from the incisal edge. Maximal torquing moments for both bracket types were observed at a height of 8 mm from the incisal edge as evident in Table 2.

**Table 2:** Torque moment values of Damon Q and Pitts 21 brackets.

S.No	Bracket height	Torque moment values of Damon Q brackets	Torque moment values of Pitts 21 brackets
1.	5 mm	9.03 Nmm	9.14 Nmm
2.	6 mm	10.13 Nmm	11.01 Nmm
3.	7 mm	12.09 Nmm	12.78 Nmm
4.	8 mm	14.11 Nmm	14.92Nmm

### 3.2 Total deformation (tooth movement) of the central incisors on engagement of a 0.019˝ x 0.025˝ SS wire into the bracket slot of a Damon Q bracket and engagement of a 0.020˝ x 0.020˝ TMA wire into the bracket slot of a Pitts 21 bracket

During torquing, the archwire is twisted inside the bracket slot which in turn generates a torque force on the tooth. These forces may also deform the bracket slot in addition to tooth movement. Bracket slot deformation under torque loads can generally be observed in brackets of poor manufacturing quality. In those situations, torque expression remains poor due to slot deformation under loading.

In this study, a twist of 1° was given to both archwires tested in order to observe torquing moments at variable bracket placement heights. Total deformation as measured by crown or root displacement, was higher in Pitts 21 than Damon Q at all tested bracket bonding heights ranging from 5 mm to 8 mm from the incisal edge. The minimum total deformation of 0.61 × 10^−6^mm and 0.41 × 10^−6^mm was observed at a bracket bonding height of 5 mm from the incisal edge for Pitts 21 and Damon Q, respectively (Fig. 2A, Fig. 2BA and B). Maximal deformation at a bonding height of 8 mm from the incisal edge was 1.23 × 10^−6^ mm for Pitts 21 in contrast to 1.11 × 10^−6^ mm for Damon Q as seen in Table 3. The increased deformation values in Pitts 21 brackets compared to Damon Q at all tested bracket bonding heights imply superior tooth movement in Pitts 21.

**Fig. 2A:**
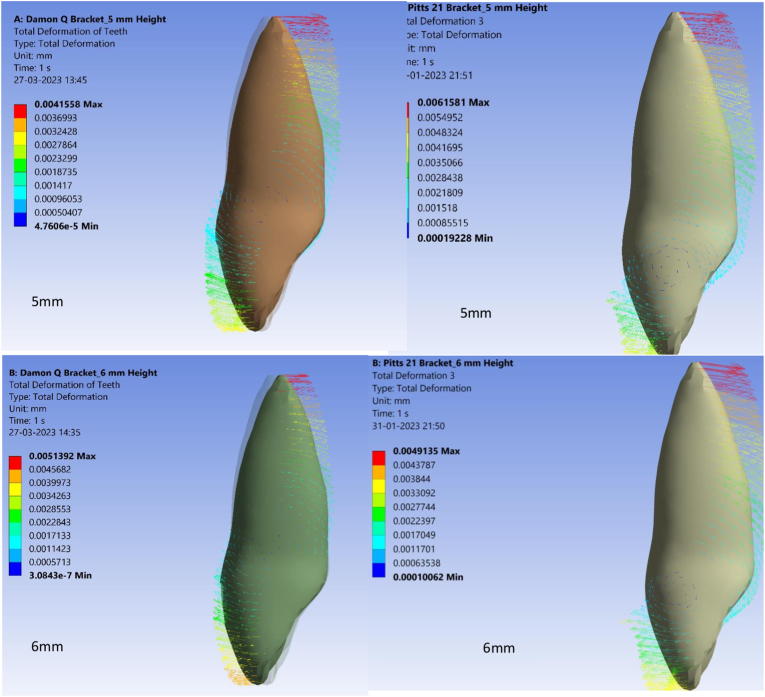
Total deformation of Damon Q and Pitts 21 brackets at 5 mm and 6 mm heights.

**Fig. 2B:**
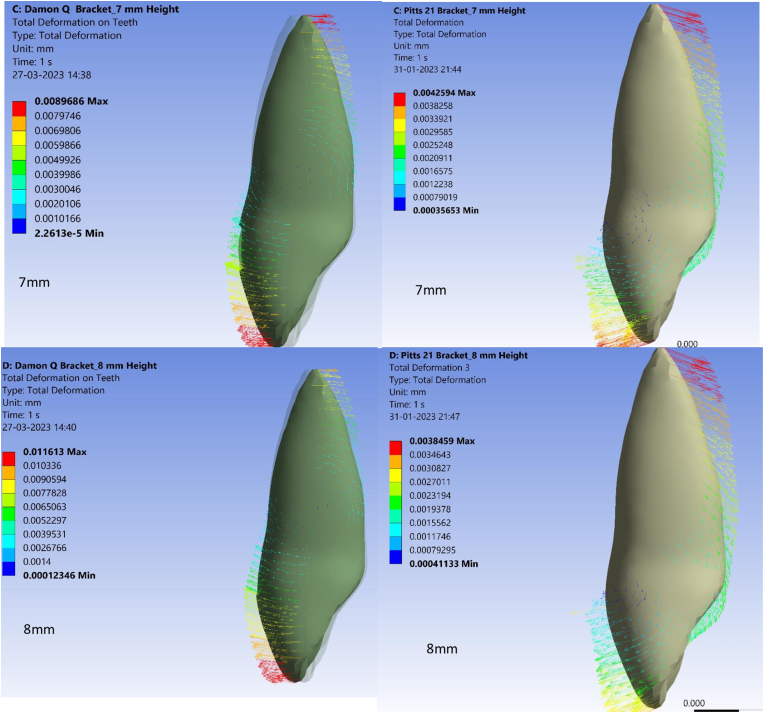
Total deformation of Damon Q and Pitts 21 brackets at 7 mm and 8 mm heights.

**Table 3:** Total deformation of Damon Q and Pitts 21 brackets.

S.No	Bracket height	Total deformation values of Damon Q	Total deformation values of Pitts 21
1.	5 mm	0.41 × 10^−6^mm	0.61 × 10^−6^mm
2.	6 mm	0.51 × 10^−6^mm	0.98 × 10^−6^mm
3.	7 mm	0.89 × 10^−6^mm	1.10 × 10^−6^mm
4.	8 mm	1.11 × 10^−6^mm	1.23 × 10^−6^mm

### 3.3 Stress generated on central incisors including roots with engagement of prescribed full-size wires into both tested bracket slots

Von Mises stress values are used in the finite element method where they allow a combination of principal stresses into an equivalent stress comparable to yield stress.^23^ Von Mises stress values also showed significant differences between Pitts 21 and Damon Q brackets at variable bracket heights (Fig. 3A, Fig. 3BA and B). Lowest Von Mises stress values were at 27.07 MPa in Damon Q brackets at a bracket height of 5 mm from the incisal edge. Highest Von Mises stress values were 36.80 MPa for Pitts 21 brackets at a bracket height of 8 mm from the incisal edge as in Table 4.

**Fig. 3A:**
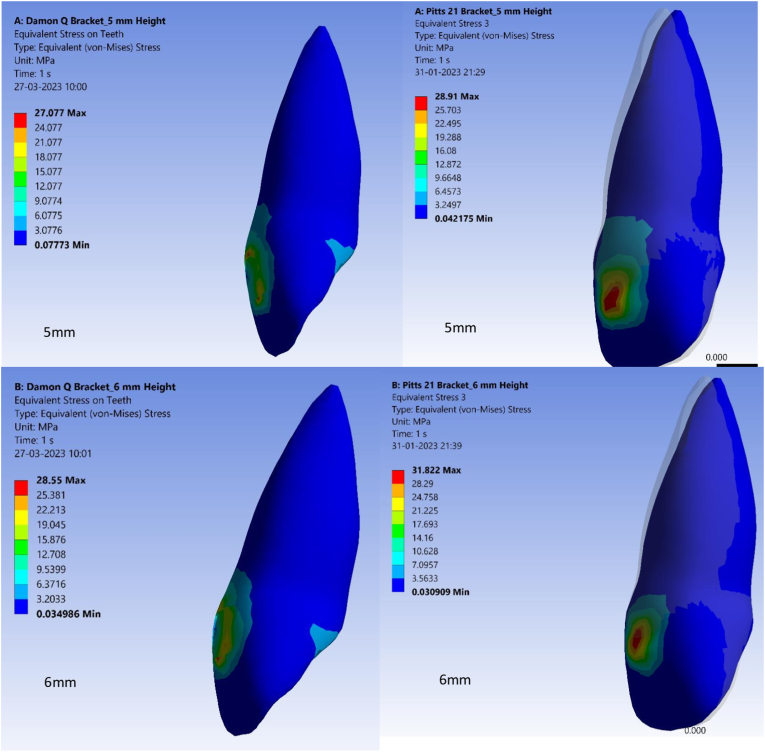
Stress on central incisor with Damon Q and Pitts 21 brackets at 5 mm and 6 mm heights.

**Fig. 3B:**
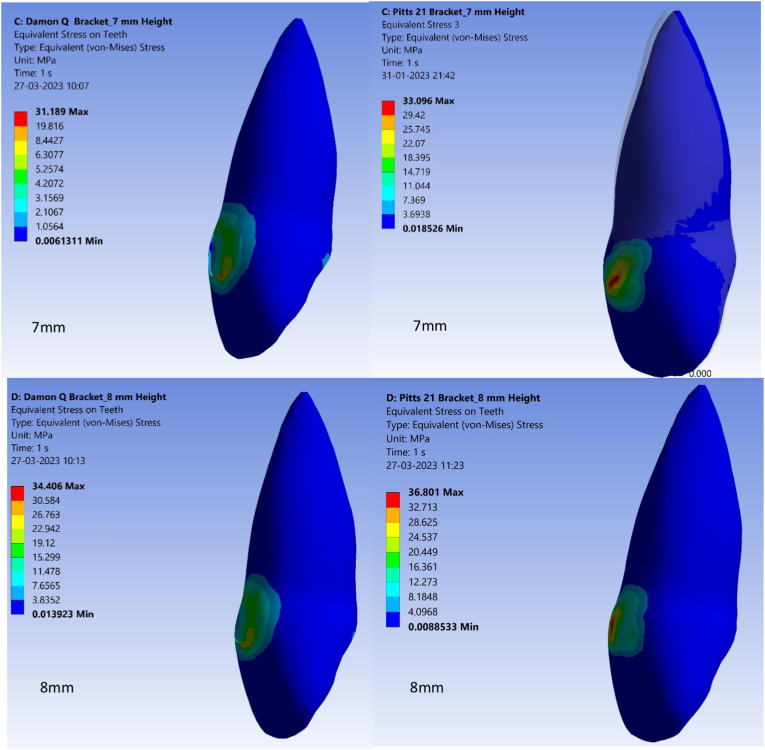
Stress on central incisor with Damon Q and Pitts 21 brackets at 7 mm and 8 mm heights.

**Table 4:** Von mises stress values of Damon Q and Pitts 21 brackets.

S.No	Bracket height	Von mises stress values of Damon Q	Von mises stress values of Pitts 21
1.	5 mm	27.07Mpa	28.91Mpa
2.	6 mm	28.55Mpa	31.82Mpa
3.	7 mm	31.18Mpa	33.09Mpa
4.	8 mm	34.40Mpa	36.80Mpa

## 4 Discussion

Bracket positioning on the maxillary incisor labial surface and expression of torque using two popular passive self-ligating brackets was analyzed in this study using the finite element method. The results indicated that displacement of the crown and root in both tested bracket types was affected by bracket positioning. The individual bracket prescription also had an effect on overall stresses seen at the level of the bracket.

Maxillary central incisors were selected since they are the most visible teeth during speech and smiling. Torque of the maxillary incisors is fundamental in establishing anterior guidance and an aesthetic smile line. Currently, there is little evidence on the torque characteristics of various bracket and archwire combinations at different heights from the incisal edge. This can be attributed to the complexity of the experimental configuration required in laboratory studies. Variation in torque expression can be due to several factors including manufacturing process, material properties, ligation methods, bracket type (conventional, self-ligating) and wire-bracket combinations. Aside from bracket prescription, bracket placement also affects the expression of desired amount of torque.^20^ As the labial contour of the crown surface varies at different heights on the tooth crown, an archwire, fully engaged in a bracket, will produce different axial inclinations at different crown heights.^24^ Batni et al. evaluated torque expression of a right permanent maxillary central incisor with varying bracket positions using PEA brackets. For all models, torque expression increased incrementally by 6° as the bracket height from the incisal edge was changed from 3 mm to 4.5 mm and 6 mm, irrespective of the crown root angulations. With increase in the crown-root angles of a tooth, the torque expression away from the incisal edge increases.^25^

In this study, Damon Q rectangular slot and Pitts 21 square slot brackets were placed at heights of 5, 6, 7 and 8 mm from the incisal edge with recommended final wires of 0.019˝ x 0.025˝ SS in Damon Q and 0.020˝ x 0.20˝ TMA in Pitts 21 brackets with a 1° wire twist incorporated in order to observe torque expression differences in both groups. Both groups showed an increase in torque values with gingival positioning of brackets with the square Pitts 21 bracket showing higher torque expression. This can be explained due to the large amount of play between the archwire and bracket slot of Damon Q where the final archwire is a 0.019˝ x 0.025˝ SS in a rectangular slot of dimension 0.022˝ x 0.028˝. The study results are in accordance with the findings of van Loenen et al. who stated that torque expression showed a consistent increase as brackets were placed more gingivally from the incisal edge. Therefore, when bonded as per the SAP (Smile Arc Protection) protocol, square brackets may permit greater torque control and allow superior incisor crown and root positioning.

Torque expression in passive rectangular slot self-ligating brackets has been a subject of debate due to the nature of the slot. The play between the bracket slot walls and the archwire effectively minimizes the moment applied in wires of cross-section smaller than the terminal. Dalstra et al. provided evidence of larger torsional play ranging from 19.8^°^ to 36.1^°^ in rectangular slot passive SLBs when compared to conventional SLBs.^26^ This results in inadequate torque expression on insertion of a final wire of prescribed size making complex wire bending mandatory in most instances. Shima et al. reported that horizontal and vertical play was significantly lesser in brackets with square slots (0.020ˮ x 0.020″) compared to rectangular slots (0.022ˮ x 0.028″). Additionally, the horizontal and vertical play ratios for the round and square wires within the square slot were approximately 1.0. Consequently, square-slotted brackets can more effectively achieve three-dimensional tooth movement, including labio-lingual and rotational tooth movements.^5^ Pitts 21 (0.021ˮx0.021″) square slot brackets on engagement of 0.020ˮx0.020″ TMA archwires show reduced play of 4^°^ in contrast to rectangular slot brackets which use 0.019ˮ x 0.025″ SS wires in 0.022ˮ x 0.028″ slots with a significant play of 11° and reduced torque expression.^14^ In view of these findings, square slot SLBs seem to enable more efficient and faster tooth movement due to superior torque expression compared to rectangular slot SLBs.Torque transmission is also limited by the material characteristics of the bracket. Deformation of bracket slot when a full size archwire is engaged, results in loss of torque and subsequent undesirable root movements (torque loss). Fischer-Brandies^27^ evaluated the effect on torque using three different sizes of stainless steel archwires in 0.018ˮ slot conventional brackets. They reported notching of the slot walls and additional widening of the bracket slot up to 0.016 mm. Papageorgiou^20^ et al. demonstrated that both bracket prescription and bracket height had a considerable effect on displacement of root apex and crown tip. Incisal bracket placement was associated with less root apex movement and gingival bracket placement produced more root apex movement. Harikrishnan^28^ et al. reported a gradual increase in slot deformation from the bottom to the top of the slot in both stainless steel and ceramic brackets. It was also evident that the mean slot deformation increases in direct proportion to increase in archwire size, slot size and angle of twist. Such slot deformation of a bracket directly impacts torque transfer to the tooth.^26^ In this study, as the brackets were positioned more gingivally, greater tooth deformation was observed for both prototypes tested. Meling^29^ et al. estimated effective bracket slot height by using a formula that described the relationship between bracket slot height, wire dimensions, wire edge bevel, and torsional play. With a torque measuring instrument, the torsional play was estimated for 10 different brackets (0.018-inch stated slot) of the same manufacturer and type. With known torsional play, wire dimensions and edge bevel, the bracket slot height could be calculated. One arch wire with known dimensions and edge bevel was used for all the measurements. Because bracket slots have a slight tapering toward the base, the effective slot height will be slightly different for a square arch wire than for rectangular archwire. Various bracket slot height in square and rectangular archwire affects torque expression. Armstrong et al.^30^ compared accuracy in bracket positioning between two techniques — localizing the centre of the clinical crown and measuring the distance from the incisal edge. Placement of brackets in the positions determined by measuring the distance from the incisal edge appears to be more accurate in the vertical dimension for the upper and lower anterior teeth. Furthermore, Pitts 21 brackets produced greater crown-root movement compared to Damon Q at all heights tested. This could be attributed to the use of square wires with minimal play in square slots as well as superior material properties. As per the manufacturer’s claims, Pitts21 brackets provide the tightest tolerances at ± .001″ versus ± .003″ for other brands. Combined with the reduced slot size of 0.021″, it could result in less deformation, thereby improving efficiency of torque transfer, evident from greater crown-root movement.

Square slot [0.018’’x 0.018’’] brackets have been developed for lingual^5^ and labial^6^ treatment in recent times to compensate for the deleterious effect of horizontal play. However, not much is known about the factors affecting torque expression in square slot brackets. The effect of arch wire material and dimension on torque expression in square brackets needs to be evaluated as there are no studies in literature at present. Also, with focus shifting towards aesthetic bonding, Smile Arc Protection protocol advocates more gingival bracket bonding heights than conventional bonding.^31^ As the square Self Ligating brackets bonded as per SAP protocol are closer to the centre of resistance of the teeth, more tooth deformation [labio-lingual tooth movement] is postulated and the same needs to be investigated systematically.

Torquing movements are also known to generate strain in the periodontal ligament at the apical third of the tooth and in bracket walls in contact with wire edges.^32^ In this study, highest von Mises stress generated in the central incisor in both bracket types were concentrated in the cervical 3rd of crown and increased as the brackets were bonded more apically. As less stresses were generated in Damon Q compared to Pitts 21, it implies better engagement of the final square archwire in a Pitts 21 bracket slot. Better slot engagement coupled with gingival bracket placement in Pitts 21 brackets may thus permit early and better control of incisor torque. The study’s depth in analyzing torque expression at various heights from the incisal edge, comparing different bracket types, and exploring stress distribution within brackets adds significant value. It addresses a specific aspect of orthodontic treatment, providing insights into the behavior of these brackets in varying scenarios. It provides comprehensive insights into how these variables impact tooth movement. Thus, it can be said that the literature has very limited evidence substantiating factors affecting torque expression in square labial brackets with square Titanium alloy wires at different bracket bonding heights.

At the same time, some limitations in the study also should be highlighted. The factors affecting tooth movement in the oral environment other than play, such as friction, tooth morphology etc. have not been considered in this study. In the present study, the adjacent teeth were not considered for analysis and it was assumed that brackets bonded on them were at the same level as the modeled tooth. Slot size in Pitts 21 system varies between anterior brackets (0.021”x 0.021″), premolar brackets (0.021”x 0.023″) and molar tubes (0.021”x 0.024″). This variation may change the torque expression as play varies. The response of PDL was not factored in, and it was assumed that the PDL possesses isotropic and elastic behavior.^24^ In Pitts 21 bracket system, 70% torque expression is achieved with 0.020”x 0.020″ Thermally Active Niti arch wire. Variation in torque expression with different arch wires was not studied.^33^ In this study, arch wire was twisted 1° before insertion into the bracket slot. The effect of different angles of torsion on torque expression have not been considered.^32^

Manufacturing and material dependent parameters such as edge form, cross-section and hardness of archwire and brackets have not been investigated. Effect of elastic deformation of bracket slot on the torque expression is not considered in this study.^28^ All these factors could have effects on study result validity and should be factored in while making clinical decisions.

## 5 Conclusions

Both Damon Q and Pitts 21 brackets exhibit increased torque expression when placed at a height of 8 mm from the edge of the maxillary central incisor compared to placements at 5, 6, and 7 mm heights. Pitts 21 brackets exhibited superior torque expression when compared to Damon Q at all heights tested. Torque moments increased as brackets were positioned more gingivally. Pitts 21 brackets generated greater torquing moments, tooth deformation and von Mises stress compared to Damon Q at all heights implying better wire-slot engagement, greater torquing moments, and tooth movement with this system.

Bracket slot design, material used, bracket height and the angle of engagement are responsible for overall torque expression characteristics in a system.
